# The Study of Structure—Analgesic Activity Relationships in a Series of 4-Hydroxy-2,2-dioxo-1*H*-2λ^6^,1-benzothiazine-3-carboxylic Acid Toluidides and Xylidides

**DOI:** 10.3390/scipharm84030497

**Published:** 2016-04-18

**Authors:** Igor V. Ukrainets, Lidiya A. Petrushova, Lyudmila V. Sidorenko, Alexandra A. Davidenko, Marina A. Duchenko

**Affiliations:** 1 Department of Pharmaceutical Chemistry, National University of Pharmacy, 53 Pushkinska St., Kharkiv 61002, Ukraine; dika-l1@ya.ru (L.A.P.); slv.ludmila@i.ua (L.V.S); 2 Department of Pharmaceutical Chemistry, N. I. Pirogov Vinnitsa National Medical University, 56 Pirogov St., Vinnitsa 21018, Ukraine; almusel@mail.ru (A.A.D.); mduchenko85@mail.ru (M.A.D.)

**Keywords:** amidation, analgesia, anilides, synthesis, pain syndrome, 4-hydroxy-2,2-dioxo-1*H*-2λ^6^,1-benzothiazine-3-carboxamides, 2,1-benzothiazines

## Abstract

In continuing the search for new analgesics among derivatives of 2,1-benzothiazines, a series of corresponding toluidides and xylidides of 4-hydroxy-2,2-dioxo-1*H*-2λ^6^,1-benzothiazine-3-carboxylic acid has been synthesized by the reaction of ethyl 4-hydroxy-2,2-dioxo-1*H*-2λ^6^,1-benzothiazine-3-carboxylate with equimolar amounts of mono- and dimethyl-substituted anilides in boiling dry xylene. Their structure has been confirmed by the data of elemental analysis, nuclear magnetic resonance (NMR) spectroscopy (^1^Н and ^13^С), as well as mass spectrometry. All compounds obtained were subjected to pharmacological screening to identify their analgesic properties. Testing was carried out in male rats using the standard model of the thermal tail-flick (tail immersion test) in parallel and in comparison with the structurally related drugs meloxicam and piroxicam. Among the substances studied, highly active oral painkillers have been found; they exceed the analgesic effect of the reference drugs using the same dose. Interesting structural and biological regularities have been described; they will be useful in further research on creating promising new analgesics based on 4-hydroxy-2,2-dioxo-1*H*-2λ^6^,1-benzothiazine-3-carboxamides.

## 1. Introduction

Pain and pain syndromes of different etiologies are the main motive causing people to seek immediate medical help. Unfortunately, doctors cannot always provide timely, effective aid to such patients, at least to make adequate analgesia the first step. Sometimes, even a rather extensive range of the known analgesics with different mechanisms of pain management appears to be powerless [1,2]. The causes are various side effects and as a consequence, numerous contraindications and limitations in practical use of analgesics [2,3,4,5,6,7]. In addition, there has recently been another serious problem—in many countries the cases of non-medical use [8] of drugs of this pharmacological group, as well as criminal violations [9,10] in the sphere of their circulation, have increased significantly. Therefore, the search for new analgesics satisfying modern ideas about the efficacy and safety of drugs, and most importantly, that are not capable of causing physical and psychological dependence, has not lost its relevance.

Interesting objects of study in this respect are derivatives of 4-hydroxy-2,2-dioxo-1*H*-2λ^6^,1-benzothiazine-3-carboxylic acids. Their lower alkyl esters [11,12] and especially hetaryl- [13,14,15,16], aryl- [17,18] and benzyl- [19] amides in experiments on animals showed a high level of analgesic properties significantly exceeding the activity of structurally related drugs of oxicam series (meloxicam and piroxicam) in the same dose.

**Figure 1:**
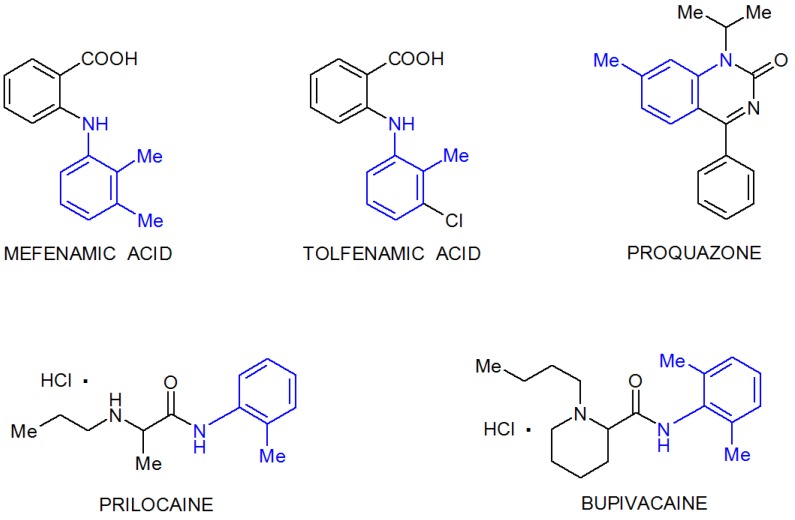
Non-narcotic analgesics containing fragments of toluidines and xylidines [1,2].

In continuing the search of potential analgesics among 4-hydroxy-2,2-dioxo-1*H*-2λ^6^,1-benzothiazine-3-carboxamides, it is quite logical and reasonable, in our opinion, to include mono- and dimethyl-substituted anilides to the compounds studied. Even a cursory inspection of the list of painkillers [1,2] which are available in medicine shows that many of them contain fragments of toluidines and xylidines in their structure (Figure 1). Thus, based on these anilines, some non-steroidal anti-inflammatory drugs with a pronounced analgesic effect (e.g., mefenamic, tolfenamic, or niflumic acids), bi- and tricyclic analgesics (proquazone and azapropazone), as well as a large series of highly active local anesthetics (lidocaine, bupivacaine, tolycaine, ropivacaine, etc.) have been created. Exactly these facts were a prerequisite for conducting our work. Its aim was to synthesize mono- and dimethyl-substituted anilidesof 4-hydroxy-2,2-dioxo-1*H*-2λ^6^,1-benzothiazine-3-carboxylic acid, study their spectral characteristics and analgesic properties, and identify the possible structural and biological regularities which can be the basis for future investigations of promising new analgesics among 2,1-benzothiazine derivatives.

## 2. Materials and Methods

### 2.1. Chemistry

#### 2.1.1. General Methods

^1^Н- and ^13^С-NMR spectra were acquired on a Varian Mercury-400 (Varian Inc., Palo Alto, CA, USA) instrument (400 and 100 MHz, respectively) in DMSO-d_6_ with tetramethylsilane as internal standard. The chemical shift values were recorded on the *δ* scale and the coupling constants (*J*) were in hertz. The following abbreviations were used in reporting spectra: s = singlet, d = doublet, t = triplet, m = multiplet. The electron impact mass spectra were recorded on a Varian 1200L mass spectrometer (Varian Inc., Walnut Creek, CA, USA) with complete scanning in the *m*/*z* range from 35 to 700 and direct sample inlet. The electron impact ionization was at 70 eV. Elemental analysis was performed on a Euro Vector EA-3000 microanalyzer (Eurovector SPA, Redavalle, Italy). Melting points were determined in a capillary using a Stuart SMP10 (Bibby Scientific Limited, Stone, Staffordshire, UK) digital melting point apparatus. The starting ethyl 4-hydroxy-2,2-dioxo-1*H*-2λ^6^,1-benzothiazine-3-carboxylate (**2**) was prepared by our previous procedure [11].

### 2.2. General Procedure for the Synthesis of *N*-aryl-4-hydroxy-2,2-dioxo-1*H*-2λ^6^,1-benzothiazine-3-carboxamides (***1a***–***h***)

A mixture of ethyl ester **2** (2.69 g, 0.01 mol), corresponding toluidine or xylidine (0.01 mol), and dry xylene (2 mL) was kept for 1 h at 150 °C on a liquid metal bath using a suitable air-cooled distilling column that allowed us to distill off the ethanol formed without removing the xylene solvent. The reaction mixture was cooled, EtOH (5 mL) was added, and the mixture was left for several hours at room temperature. The crystalline amide 1 precipitate was filtered off, washed with cold EtOH, dried, and recrystallized from the suitable solvent. Toluidides and xylidides **1a**–**h** are colorless or white with yellowish tinted crystals.

*4-Hydroxy-*N*-(2-methylphenyl)-2,2-dioxo-1*Н*-2λ^6^,1-benzothiazine-3-carboxamide* (**1a**).Yield: 88%; melting point (mp) 206–208 °C (methylene chloride); ^1^H-NMR (400 MHz, DMSO-d_6_): δ 15.53 (br. s, 1H, 4-ОН), 12.22 (br. s, 1H, SO_2_NH), 9.35 (s, 1Н, CONH), 8.00 (d, 1Н, *J* = 8.3 Hz, Н-5), 7.88 (d, 1H, *J* = 7.9 Hz, Н-6′), 7.62 (t, 1Н, *J* = 7.7 Hz, Н-7), 7.30–7.19 (m, 4Н, Н-6,8,3′,4′), 7.12 (t, 1Н, *J* = 7.4 Hz, Н-5′), 2.35 (s, 3Н, CH_3_). ^13^C-NMR (100 MHz, DMSO-d_6_): δ 168.8 (С-ОН), 164.6 (С=О), 137.7, 136.4, 134.4, 130.3, 126.3, 126.0, 125.6, 123.6, 123.0, 119.4, 118.3, 116.5, 103.6 (C-3), 17.2 (CH_3_). Mass Spectrum (MS) (*m*/*z*, %): 330 [M]^+^ (2.2), 223 (1.4), 197 (6.0), 133 (51.5), 107 (91.1), 106 (60.5), 105 (35.5), 104 (100), 92 (44.9), 78 (70.3), 77 (65.5). Analytical Calculated (Anal. Calcd.) for C_16_H_14_N_2_O_4_S: C, 58.17; H, 4.27; N, 8.48; S 9.71%. Found: C, 58.13; H, 4.20; N, 8.53; S 9.79%.

*4-Hydroxy-*N*-(3-methylphenyl)-2,2-dioxo-1*Н*-2λ^6^,1-benzothiazine-3-carboxamide* (**1b**). Yield: 92%; mp 183–185 °C (methylenechloride); ^1^H-NMR (400 MHz, DMSO-d_6_): δ 15.44 (br. s, 1H, 4-OH), 12.24 (br. s, 1H, SO_2_NH), 9.41 (s, 1H, CONH), 7.99 (d, 1H, *J* = 8.0 Hz, H-5), 7.61 (t, 1H, *J* = 7.8 Hz, H-7), 7.42 (d, 1H, *J* = 7.8 Hz, H-6′), 7.35 (s, 1H, H-2′), 7.29-7.23 (m, 2H, H-6,5′), 7.20 (d, 1H, *J* = 8.0 Hz, H-8), 6.99 (d, 1H, *J* = 7.6 Hz, H-4′), 2.37 (s, 3H, Me). ^13^C-NMR (100 MHz, DMSO-d_6_): δ168.3 (С-ОН), 164.5 (С=О), 138.3, 136.6, 134.3, 128.2, 126.1, 125.6, 125.1, 123.0, 121.5, 119.7, 118.2, 116.3, 104.2 (C-3), 20.9 (CH_3_). MS (*m/z*, %): 330 [M]^+^ (6.7), 223 (1.1), 197 (2.8), 133 (28.5), 119 (39.3), 107 (100), 106 (65.2), 105 (14.3), 104 (39.6), 92 (28.7), 78 (51.9), 77 (62.6). Anal. Calcd. for C_16_H_14_N_2_O_4_S: C, 58.17; H, 4.27; N, 8.48; S 9.71%. Found: C, 58.25; H, 4.33; N, 8.41; S 9.76%.

*4-Hydroxy-*N*-(4-methylphenyl)-2,2-dioxo-1*Н*-2λ^6^,1-benzothiazine-3-carboxamide* (**1c**). Yield: 95%; mp 217–219 °C (methylenechloride); ^1^H-NMR (400 MHz, DMSO-d_6_): δ 15.47 (br. s, 1H, 4-OH), 12.23 (br. s, 1H, SO_2_NH), 9.38 (s, 1H, CONH), 7.99 (d, 1H, *J* = 8.1 Hz, H-5), 7.60 (t, 1H, *J* = 7.7 Hz, H-7), 7.46 (d, 2H, *J* = 8.4 Hz, H-2′,6′), 7.25 (t, 1Н, *J* = 7.7 Hz, Н-6), 7.20 (d, 1H, *J* = 8.1 Hz, H-8), 7.16 (d, 2H, *J* = 8.4 Hz, H-3′,5′), 2.34 (s, 3H, Me). ^13^C-NMR (100 MHz, DMSO-d_6_): δ167.9 (С-ОН), 163.8 (С=О), 136.7, 134.5, 129.3 (C-3′,5′), 128.2, 126.1, 123.4, 122.9, 121.3 (C-2′,6′), 119.0, 118.1, 104.0 (C-3), 20.2 (CH_3_). MS (*m*/*z*, %): 330 [M]^+^ (4.5), 223 (2.5), 197 (1.3), 133 (77.3), 119 (39.1), 107 (99.5), 106 (83.9), 105 (17.9), 104 (76.4), 92 (56.7), 78 (45.1), 77 (100). Anal. Calcd. for C_16_H_14_N_2_O_4_S: C, 58.17; H, 4.27; N, 8.48; S 9.71%. Found: C, 58.26; H, 4.35; N, 8.40; S 9.65%.

N*-(2-Bromo-4-methylphenyl)-4-hydroxy-2,2-dioxo-1*Н*-2λ^6^,1-benzothiazine-3-carboxamide (***1d**). Yield: 84%; mp 231–233 °C (ethylacetate); ^1^H-NMR (400 MHz, DMSO-d_6_): δ 15.29 (br. s, 1H, 4-OH), 12.25 (br. s, 1H, SO_2_NH), 9.65 (s, 1H, CONH), 8.05 (d, 1H, *J* = 7.8 Hz, H-6′), 7.99 (d, 1H, *J* = 8.0 Hz, H-5), 7.63 (t, 1H, *J* = 7.7 Hz, H-7), 7.49 (s, 1H, H-3′), 7.28 (t, 1H, *J* = 7.6 Hz, H-6), 7.22 (d, 1H, *J* = 8.0 Hz, H-8), 7.19 (d, 1H, *J* = 7.8 Hz, H-5′), 2.35 (s, 3H, Me). ^13^C-NMR (100 MHz, DMSO-d_6_): δ169.2 (С-ОН), 164.4 (С=О), 136.1, 134.5, 132.8, 131.9, 128.8, 127.7, 126.2, 124.4, 123.1, 122.8, 118.5, 118.1, 103.6 (C-3), 19.8 (CH_3_). MS (*m*/*z*, %): 408/410 [M]^+^ (4.7/4.2), 329 (11.3), 223 (1.3), 211/213 (11.8/20.6), 197 (4.3), 185/187 (99.5/97.7), 119 (28.1), 107 (41.2), 106 (87.5), 105 (15.0), 104 (59.9), 92 (38.0), 78 (45.9), 77 (100). Anal. Calcd. for C_16_H_13_BrN_2_O_4_S: C, 46.96; H, 3.20; N, 6.84; S 7.83%. Found: C, 47.05; H, 3.26; N, 6.75; S 7.76%.

N*-(2,3-Dimethylphenyl)-4-hydroxy-2,2-dioxo-1*Н*-2λ^6^,1-benzothiazine-3-carboxamide* (**1e**). Yield: 90%; mp 212–214 °C (ethanol); ^1^H-NMR (400 MHz, DMSO-d_6_): δ 15.63 (br. s, 1H, 4-OH), 12.19 (br. s, 1H, SO_2_NH), 9.28 (s, 1H, CONH), 7.99 (d, 1H, *J* = 8.0 Hz, H-5), 7.61 (t, 1H, *J* = 7.8 Hz, H-7), 7.56 (d, 1H, *J* = 8.0 Hz, H-6′), 7.27 (t, 1H, J = 7.8 Hz, H-6), 7.22 (d, 1H, *J* = 8.3 Hz, H-8), 7.10 (t, 1H, *J* = 7.7 Hz, H-5′), 7.05 (d, 1H, *J* = 7.5 Hz, H-4′), 2.35 (s, 3H, Me), 2.23 (s, 3H, Me). ^13^C-NMR (100 MHz, DMSO-d_6_): δ168.8 (С-ОН), 164.7 (С=О), 137.1, 134.5, 129.8, 128.1, 127.5, 126.1, 125.4, 123.0, 122.3, 119.2, 118.1, 116.5, 103.4 (C-3), 19.9 (CH_3_), 13.4 (CH_3_). MS (*m*/*z*, %): 344 [M]^+^ (2.1), 223 (1.4), 197 (3.3), 147 (16.5), 133 (2.3), 121 (100), 120 (56.4), 119 (51.6), 107 (26.4), 106 (60.3), 105 (14.6), 104 (31.0), 92 (48.1), 78 (50.8), 77 (80.0). Anal. Calcd. for C_17_H_16_N_2_O_4_S: C, 59.29; H, 4.68; N, 8.13; S 9.31%. Found: C, 59.22; H, 4.63; N, 8.20; S 9.25%.

N*-(2,4-Dimethylphenyl)-4-hydroxy-2,2-dioxo-1*Н*-2λ^6^,1-benzothiazine-3-carboxamide* (**1f**). Yield: 87%; mp 195–197 °C (methylenechloride-methanol, 1:1); ^1^H-NMR (400 MHz, DMSO-d_6_): δ 15.59 (br. s, 1H, 4-OH), 12.20 (br. s, 1H, SO_2_NH), 9.26 (s, 1H, CONH), 7.99 (d, 1H, *J* = 8.1 Hz, H-5), 7.71 (d, 1H, *J* = 8.3 Hz, H-6′), 7.62 (t, 1H, *J* = 7.7 Hz, H-7), 7.26 (t, 1H, J = 7.7 Hz, H-6), 7.21 (d, 1H, *J* = 8.3 Hz, H-8), 7.07 (s, 1H, H-3′), 7.02 (d, 1H, *J* = 8.3 Hz, H-5′), 2.32 (s, 3H, Me), 2.30 (s, 3H, Me). ^13^C-NMR (100 MHz, DMSO-d_6_): δ168.9 (С-ОН), 164.4 (С=О), 137.8, 136.1, 133.7, 131.5, 130.7, 127.8, 126.5, 126.0, 123.1, 122.6, 122.1, 117.8, 103.7 (C-3), 20.3 (CH_3_), 17.3 (CH_3_). MS (*m/z*, %): 344 [M]^+^ (6.5), 223 (2.5), 197 (4.2), 147 (16.2), 133 (4.8), 121 (100), 120 (53.1), 119 (21.7), 107 (20.4), 106 (61.9), 105 (17.4), 104 (35.1), 92 (28.5), 78 (28.3), 77 (60.5). Anal. Calcd. for C_17_H_16_N_2_O_4_S: C, 59.29; H, 4.68; N, 8.13; S 9.31%. Found: C, 59.35; H, 4.77; N, 8.05; S 9.24%.

N*-(2,5-Dimethylphenyl)-4-hydroxy-2,2-dioxo-1*Н*-2λ^6^,1-benzothiazine-3-carboxamide* (**1g**). Yield: 90%; mp 220–222 °C (ethanol); ^1^H-NMR (400 MHz, DMSO-d_6_): δ 15.55 (br. s, 1H, 4-OH), 12.21 (br. s, 1H, SO_2_NH), 9.29 (s, 1H, CONH), 8.00 (d, 1H, *J* = 8.1 Hz, H-5), 7.71 (s, 1H, H-6′), 7.62 (t, 1H, *J* = 7.8 Hz, H-7), 7.29 (t, 1H, *J* = 7.8 Hz, H-6), 7.22 (d, 1H, *J* = 8.1 Hz, H-8), 7.12 (d, 1H, *J* = 7.7 Hz, H-4′), 6.93 (d, 1H, *J* = 7.7 Hz, H-3′), 2.35 (s, 3H, Me), 2.30 (s, 3H, Me). ^13^C-NMR (100 MHz, DMSO-d_6_): δ168.7 (С-ОН), 164.3 (С=О), 137.7, 135.6, 134.4, 130.2, 128.1, 126.5, 126.0, 123.9, 123.1, 119.5, 118.2, 116.6, 103.5 (C-3), 20.4 (CH_3_), 16.9 (CH_3_). MS (*m/z*, %): 344 [M]^+^ (7.9), 223 (1.8), 197 (23.1), 147 (86.7), 133 (14.1), 121 (100), 120 (52.7), 119 (80.0), 107 (15.5), 106 (69.3), 105 (42.5), 104 (71.9), 92 (36.6), 78 (75.5), 77 (94.2). Anal. Calcd. for C_17_H_16_N_2_O_4_S: C, 59.29; H, 4.68; N, 8.13; S 9.31%. Found: C, 59.34; H, 4.61; N, 8.22; S 9.22%.

N*-(2,6-Dimethylphenyl)-4-hydroxy-2,2-dioxo-1*Н*-2λ^6^,1-benzothiazine-3-carboxamide* (**1h**). Yield: 85%; mp 236–238 °C (ethanol); ^1^H-NMR (400 MHz, DMSO-d_6_): δ 15.74 (br. s, 1H, 4-OH), 12.11 (br. s, 1H, SO_2_NH), 8.97 (s, 1H, CONH), 7.97 (d, 1H, *J* = 8.1 Hz, H-5), 7.62 (t, 1H, *J* = 7.7 Hz, H-7), 7.26 (t, 1H, *J* = 7.7 Hz, H-6), 7.21 (d, 1H, *J* = 8.2 Hz, H-8), 7.17–7.09 (m, 3H, H-3′,4′,5′), 2.27 (s, 6H, 2 × Me). ^13^C-NMR (100 MHz, DMSO-d_6_): δ168.9 (С-ОН), 165.0 (С=О), 135.1, 134.5, 132.9, 127.7 (C-3′,5′), 127.4 (C-2′,6′), 126.0, 123.0, 119.5, 118.1, 116.4, 103.3 (C-3), 17.8 (2 × CH_3_). MS (*m/z*, %): 344 [M]^+^ (8.0), 223 (2.0), 197 (7.4), 147 (3.7), 133 (3.1), 121 (100), 120 (36.0), 119 (23.7), 107 (4.4), 106 (28.7), 105 (9.6), 104 (17.5), 92 (21.9), 78 (16.7), 77 (26.5). Anal. Calcd. for C_17_H_16_N_2_O_4_S: C, 59.29; H, 4.68; N, 8.13; S 9.31%. Found: C, 59.20; H, 4.62; N, 8.09; S 9.24%.

### 2.3. Pharmacology

#### 2.3.1. Analgesic Test

All biological experiments were carried out in full accordance with the European Convention on the Protection of Vertebrate Animals Used for Experimental and Other Scientific Purposes and the Ukrainian Law No. 3447-IV “On protection of animals from severe treatment” (2006) (project ID 3410U14 approved October 15, 2015). The analgesic properties of toluidides and xylidides **1a**–**h** were studied compared to piroxicam (Jenapharm, Jena, Germany) and meloxicam (Boehringer Ingelheim, Ingelheim am Rhein, Germany) being similar to the structure on the model of the thermal tail-flick procedure in white rats (tail immersion test) [20].This allowed us to judge the central effect on the nociceptive system. For this purpose, the rat’s tail tip was immersed in a water bath heated to 54 °C, and the latent period of the tail withdrawal (immersion) expressed in seconds was determined. The analgesic effect (in %) was assessed by the change of the latent period in1 hour after introduction of the test substances and reference drugs. Seven experimental animals were used to obtain statistically reliable results (the significance level of the confidence interval accepted in this work was *p* ≤ 0.05) in testing each of the toluidides and xylidides **1a**–**h**, reference drugs, and control. All substances under research, piroxicam, and meloxicam were introduced orally in the form of fine aqueous suspensions stabilized with Tween80 in the dose of 20 mg/kg. The animals in the control group received an equivalent amount of water with Tween80.

## 3. Results and Discussion

### 3.1. Chemistry

**Scheme 1:**
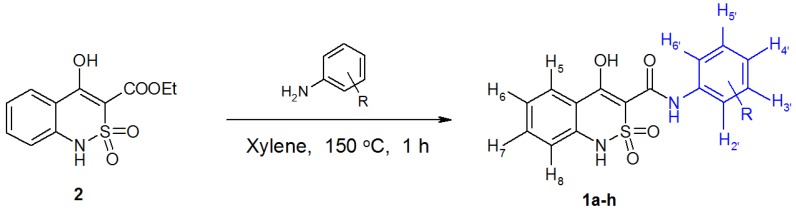
Synthesis of 4-hydroxy-2,2-dioxo-1*H*-2λ^6^,1-benzothiazine-3-carboxylic acid toluidides and xylidides**1**. **1**: **a**R = 2-Me; **b**R = 3-Me; **c**R = 4-Me; **d**R = 2-Br-4-Me; **e**R = 2,3-(Me)_2_; **f**R = 2,4-(Me)_2_; **g**R = 2,5-(Me)_2_; **h**R = 2,6-(Me)_2_.

The synthesis of target compounds of the research has been carried out by the reaction of ethyl 4-hydroxy-2,2-dioxo-1*H*-2λ^6^,1-benzothiazine-3-carboxylate (**2**) and the corresponding toluidines or xylidines in boiling dry xylene (Scheme 1). This method, that is simple to perform, makes it easy to obtain toluidides and xylidides of 4-hydroxy-2,2-dioxo-1*H*-2λ^6^,1-benzothiazine-3-carboxylic acid **1a**–**h** with good yields and purity.

All toluidides and xylidides (**1a**–**h**) synthesized in such a way are colorless or white crystalline substances with a yellowish tint and a narrow interval of melting points (see Materials and Methods). At room temperature they are soluble in dimethylformamide (DMF) and dimethyl sulfoxide (DMSO), slightly soluble in lower alkyl alcohols, and practically insoluble in water. To confirm their structure, elemental analyses by nuclear magnetic resonance (NMR; ^1^Н and ^13^С) as well as mass spectrometry were used.

The ^1^Н-NMR spectra clearly show that all toluidides and xylidides of 4-hydroxy-2,2-dioxo-1*H*-2λ^6^,1-benzothiazine-3-carboxylic acid (**1а**–**h**) exist in 4-ОНform in the solution of DMSO-d_6_. This is evidenced by the singlet signals of hydroxyl protons in a weak field, which is typical for enols (16–15 ppm). A noticeable broadening of these signals can be explained by the pronounced acidic properties of 4-ОНgroups in 4-hydroxy-2,2-dioxo-1*H*-2λ^6^,1-benzothiazine-3-carboxamides [21] and, as a consequence, by their propensity for proton exchange, formation of intra- and intermolecular hydrogen bonds, as well as association with solvents. The same can be said about cyclic sulfonamide groups (using X-ray diffraction analysis we have confirmed that the derivatives of 4-hydroxy-2,2-dioxo-1*H*-2λ^6^,1-benzothiazine-3-carboxylic acids that are unsubstituted in position 1 can form salts with amines in the cyclic sulfonamide group and the 4-ОНgroup remains free)—singlet signals of their protons in all cases are also broad.

**Figure 2:**
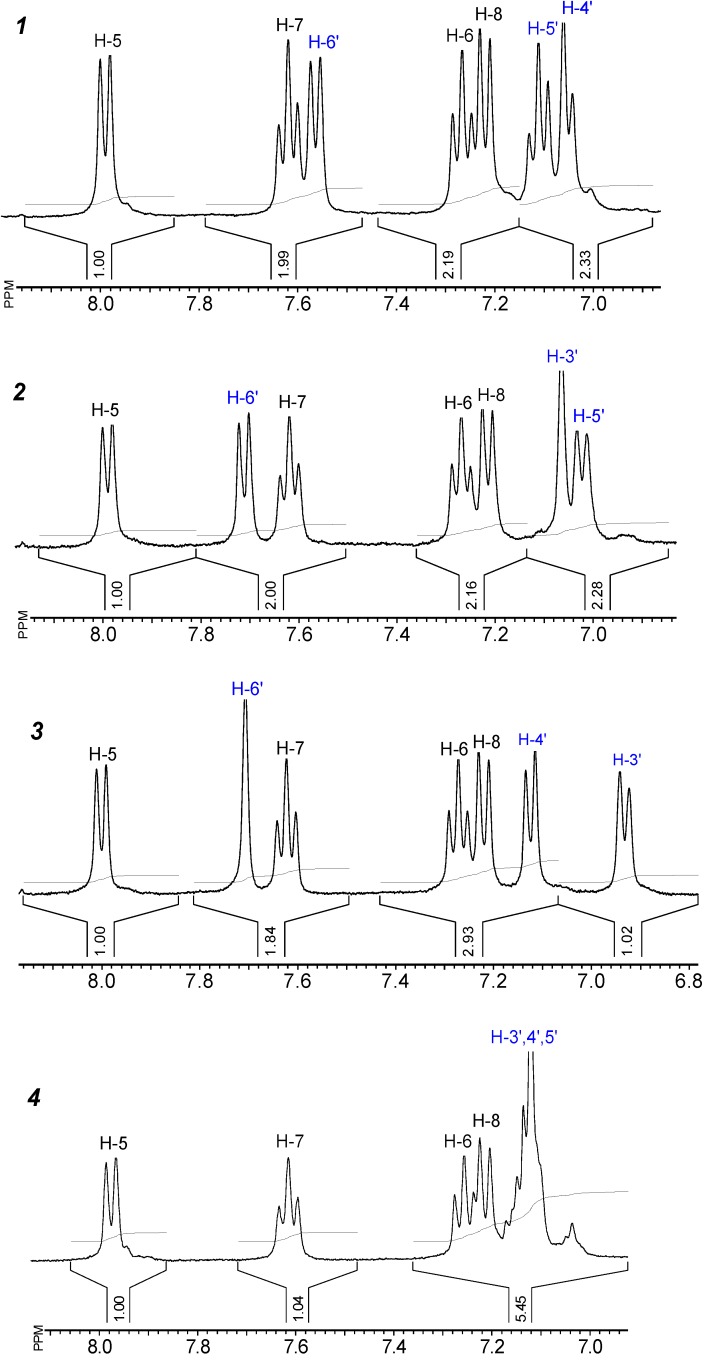
Fragments of the ^1^H-NMR spectra (signals of aromatic protons) of xylidides: *1*—2,3-(Me)_2_ (**1e**); *2*—2,4-(Me)_2_ (**1f**); *3*—2,5-(Me)_2_ (**1g**); *4*—2,6-(Me)_2_ (**1h**).

In the “aromatic” region of the ^1^Н-NMR spectra oftoluidides and xylidides **1а**–**h**, signals of seven or even eight aromatic protons are simultaneously focused on very short segments of about 1 ppm. However, the coincidence of the resonant frequencies is very rare; most of the aromatic protons can be easily identified by chemical shifts and multiplicity of their signals, and if necessary, by taking into account the values of the constants of spin–spin interactions (see Materials and Methods). In this respect, the^1^Н-NMR spectra of xylidides **1е**–**h** are especially interesting and significant (Figure 2). For example, in the case of 2,3-, 2,4- and 2,5-dimethyl-substituted isomers, the classical ^1^Н-NMRspectra are observed—the signals of all aromatic protons are well-resolved; it allows us to reliably determine the location of methyl groups in the aromatic ring. But the picture is highly distorted only in the spectrum of 2,6-dimethylanilide **1h**: now the signals of aromatic protons of only thebenzothiazine cycle are clearly specified, while all protons of the anilide fragment are presented by one common multiplet. This effect is possible under the condition when any steric obstacles prevent conjugation. Among all xylidides **1е**–**h** the aromatic nucleus cannot be located in the plane of the amide fragment in the presence of at least two methyl groups in adjacent positions, i.e., 2 and 6. Only this location of the methyl substituents leads to the absence of conjugation and, therefore, reduction of differences in the chemical shifts of the protons of the *N*-aryl fragment. In other words, the coincidence of resonant frequencies of aromatic protons of the anilidemoiety of the molecule observed in the experiment indicates that the sample under research is exactly 2,6-dimethyl-substituted anilide **1h**.

The ^13^C-NMRspectra of toluidides and xylidides **1а**–**h** gave important and useful information about their structure, uniquely determining the number of carbon atoms in the substances studied. The position of all signals in this case is in the range of shifts that are typical for their chemical environment (see Materials and Methods).

**Scheme 2:**
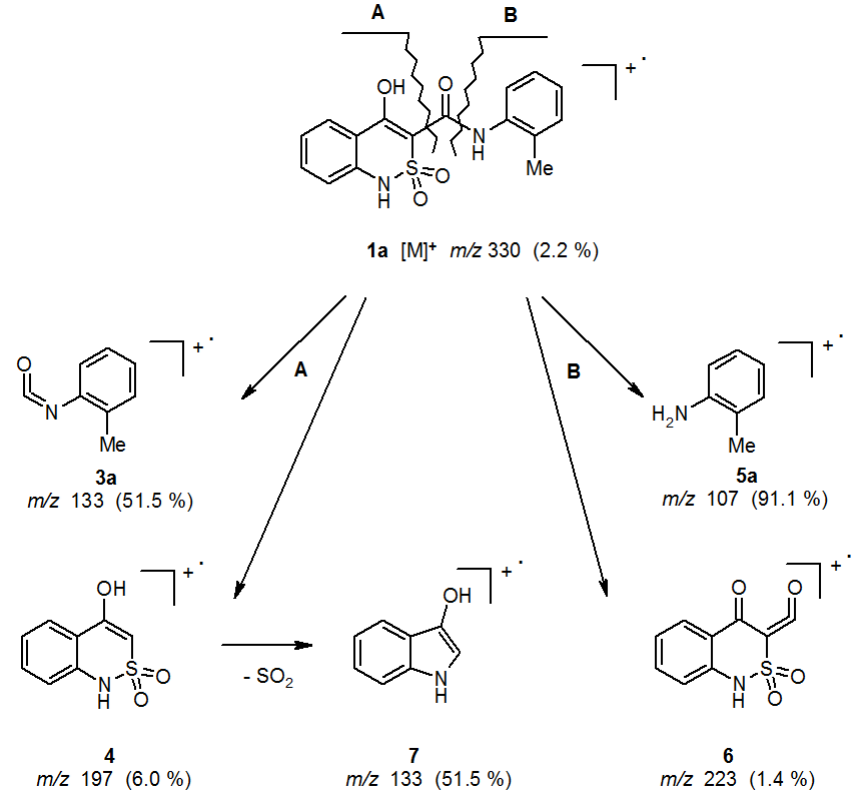
A typical example of the mass spectrometric behavior of 4-hydroxy-2,2-dioxo-1*H*-2λ^6^,1-benzothiazine-3-carboxylic acid toluidides and xylidides.

The mass spectrometric behavior of all toluidides and xylidides (**1а**–**h)** synthesized was similar. The instability of the compounds under research immediately attracts attention—all of them are quite easily destroyed under ionization by electron impact; as a result, the peak intensity of molecular ions is less than 10% for all of the examples. A typical example of 2-toluidide **1а** below shows (Scheme 2) that the class of the compounds studied is characterized by two directions of the primaryfragmentation ofmolecular ions: breaking of the heterocycle-3-anilide substituent bond (pathway А) or destruction of the acyclic amide bond (pathway В).

The existence of toluidides and xylidides **1а**–**h** in the 4-ОН form is confirmed by the fact that noneof them are subjected to the primary elimination of SO_2_ under the influence of electron impact ionization. This is quite a specific process, which is common for many cyclic sulfoderivatives [22,23]. In the case of 4-hydroxy-2,2-dioxo-1*H*-2λ^6^,1-benzothiazine-3-carboxamides, it is characteristic only for those compounds that exist in the form of internal salts (4-olates) [14,16]. When the mass spectra of 4-hydroxy derivatives (i.e., while transferring into the gas phase) are registered, sulfur dioxide extrusion occurs in destruction of the fragment ions only, for example benzothiazine **4**. As a rule, the peak intensity of hydroxyindole **7** with *m/z* 133 formed in the process of this transformation is extremely low and rarely exceeds 10%. The exception is toluidides **1а**–**с**, and it is only because of the fact that in their mass spectra, the weak peaks of hydroxyindole **7** overlap the much more powerful peaks of isocyanates of type **3а** with the same *m/z* 133 (Scheme 2).

### 3.2. Evaluationof the Analgesic Activity

The results of the study of the analgesic activity of all 4-hydroxy-2,2-dioxo-1*H*-2λ^6^,1-benzothiazine-3-carboxylic acid toluidides and xylidides (**1а**–**h**) synthesized are given in Table 1. A comparative analysis of these data with the values of the similar toluidides and xylidides of 4-hydroxy-1-methyl-2,2-dioxo-1*H*-2λ^6^,1-benzothiazine-3-carboxylic acid previously tested in the same conditions [18] shows that removal of the 1-*N*-methyl substituent affects biological properties in a different way. So for example, earlier in the group of toluidides, the following order of attenuation of the analgesic effect depending on the location of the methyl group in the anilide fragment was observed: 2-Me > 4-Me >> 3-Me. But after transferring to their analogs **1а**–**с** unsubstituted in position 1, the picture changed to the opposite one: 3-Me >> 4-Me > 2-Me, and *meta-*isomer **1b** became the most active. In the case of 2-bromo-4-methylphenyl derivatives, the presence or absence of a methyl substituent at the nitrogen atom of the benzothiazine nucleus practically should have no effect on their analgesic activity. However, the modification performed by us affected the group of xylididesquite positively—all compounds **1e**–**h** without exception appeared to be highly active analgesics; moreover, the position of methyl groups in theanilide fragment had little effect on the strength of the biological effect.

## 4. Conclusions

The article presents a group of new 4-hydroxy-2,2-dioxo-1*H*-2λ^6^,1-benzothiazine-3-carboxylic acid toluidides and xylidides without substituents at the nitrogen atom of the benzothiazine cycle. The spectral characteristics of the substances synthesized have been studied. The specificity of the NMR spectra and peculiarities of the mass-spectrometric behavior confirming their existence in the 4-hydroxy form are discussed. All toluidides and xylidides obtained were subjected to pharmacological screening; as a result, compounds with high analgesic activity exceedingpiroxicamand meloxicam have been found. A comparative analysis of the biological properties of 4-hydroxy-2,2-dioxo-1*H*-2λ^6^,1-benzothiazine-3-carboxylic acid toluidides and xylidides and their 1-*N*-methyl substituted analogues has shown that position 1, that is readily subjected to modification, can be used effectively for purposeful improvement of pharmaceutical and/or pharmacological properties of compounds of the class studied.

## Figures and Tables

**Table 1:**
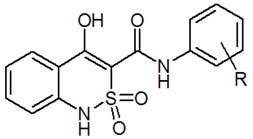
The Analgesic Activity of Toluidides and Xylidides **1a**–**h**, and Reference Drugs.

Entry	Product	R	Latent Period in 1 h after Introduction of the Compounds (s) ^a^	Change of the Latent Period, Compared to Control (%) ^b^
1	**1a**	2-Me	3.28 ± 0.13	+4.7 (+88.6)
2	**1b**	3-Me	4.19 ± 0.17	+33.3 (+7.6)
3	**1c**	4-Me	3.40 ± 0.11	+8.3 (+61.1)
4	**1d**	2-Br-4-Me	3.64 ± 0.12	+16.0 (+12.8)
5	**1e**	2,3-(Me)_2_	5.39 ± 0.15	+71.8 (+1.9)
6	**1f**	2,4-(Me)_2_	5.35 ± 0.16	+70.3 (+68.6)
7	**1g**	2,5-(Me)_2_	5.14± 0.17	+64.0 (+39.7)
8	**1h**	2,6-(Me)_2_	4.86 ± 0.16	+54.7 (+5.9)
9	Meloxicam	–	4.91± 0.17	+56.3
10	Piroxicam	–	3.96 ± 0.15	+26.1
11	Control	–	3.14± 0.14	–

^a^ All results from biological tests were analyzed statistically using Student’s *t*-test. Effects were regarded as statistically significant at *p* ≤ 0.05. ^b^ The data on the analgesic activity of the corresponding toluidides and xylidides of 4-hydroxy-1-methyl-2,2-dioxo-1*H*-2λ^6^,1-benzothiazine-3-carboxylic acid [18] are given inparentheses.
